# Gender differences affect the location of the patellar tendon attachment site for tibial rotational alignment in total knee arthroplasty

**DOI:** 10.1186/s13018-022-03248-5

**Published:** 2022-07-26

**Authors:** Le-Shu Zhang, Jin-Cheng Zhang, Hang Zhou, Qiang Zhang, Xiang-Yang Chen, Shuo Feng

**Affiliations:** Department of Orthopedic Surgery, Affiliated Hospital of Xuzhou Medical University, 99 Huaihai Road, Xuzhou, 221002 Jiangsu China

**Keywords:** Total knee arthroplasty, Rotational alignment, Computer simulation, Tibial component

## Abstract

**Purpose:**

This study was carried out to investigate the accuracy of referring different locations of the patellar tendon attachment site and the geometrical center of the osteotomy surface for tibial rotational alignment and observe the influences of gender differences on the results.

**Methods:**

Computed tomography scans of 135 osteoarthritis patients (82 females and 53 males) with varus deformity was obtained to reconstruct three-dimensional (3D) models preoperatively. The medial boundary, medial one-sixth, and medial one-third of the patellar tendon attachment site were marked on the tibia. These points were projected on the tibial osteotomy plane and connected to the geometrical center (GC) of the osteotomy plane or the middle of the posterior cruciate ligament (PCL) to construct six tibial rotational axes (Akagi line, MBPT, MSPT1, MSPT2, MTPT1 and MTPT2). The mismatch angle between the vertical line of the SEA projected on the proximal tibial osteotomy surface and six different reference axes was measured. In additional, the effect of gender differences on rotational alignment for tibial component were assessed.

**Results:**

Relative to the SEA, rotational mismatch angles were − 1.8° ± 5.1° (Akagi line), − 2.5° ± 5.3° (MBPT), 2.8° ± 5.3° (MSPT1), 4.5° ± 5.4° (MSPT2), 7.3° ± 5.4° (MTPT1), and 11.6° ± 5.8° (MTPT2) for different tibial rotational axes in all patients. All measurements differed significantly between the male and female. The tibial rotational axes with the least mean absolute deviation for the female or male were Akagi line or MSPT, respectively. There was no significant difference in whether the GC of the osteotomy surface or the midpoint of PCL termination was chosen as the posterior anatomical landmark when the medial boundary or medial one-sixth point of the patellar tendon attachment site was selected as the anterior anatomical landmark.

**Conclusion:**

When referring patellar tendon attachment site as anterior anatomical landmarks for tibial rotational alignment, the influence of gender difference on the accuracy needs to be taken into account. The geometric center of the tibial osteotomy plane can be used as a substitute for the middle of the PCL termination when reference the medial boundary or medial one-sixth of the patellar tendon attachment site.

**Supplementary Information:**

The online version contains supplementary material available at 10.1186/s13018-022-03248-5.

## Introduction

Total knee arthroplasty (TKA) is known as the most common surgical treatment for knee osteoarthritis (OA). It is effective to improve the quality of life of patients. However, it is still reported that 12.7% of patients failed to achieve satisfactory surgical treatments after primary total knee arthroplasty, mainly in terms of poor postoperative pain relief, limitation in the range of knee motion, and instability of the knees [3, 8]. There are a considerable number of relevant factors that influence the success of total knee arthroplasty, of which the correct rotational alignment of tibial components is one of the key factors [4]. Some previous studies have reported that malposition of tibial components in the horizontal plane can be the cause of patellar complications [7], unexplained knee pain [6, 17], knee stiffness [1, 5], and polyethylene wear [9, 14].

Various anatomical landmarks have been applied for rotational alignment in primary TKA. The prominent point of lateral femoral epicondyle and the medial femoral epicondylar sulcus have been connected to establish the surgical transepicondylar axis (SEA) for femoral rotational alignment. It is not only the functional flexion–extension axis of the knee [2, 18] but also the femoral rotational axis [10]. Installing femoral and tibial component in accordance with SEA is consistent with biomechanical characteristics [12, 22]. On the tibial side, due to the inability of the surgeons to locate the SEA directly on the proximal tibial osteotomy surface, the researchers have attempted to establish optimal tibial rotational axes using appropriate intra- or extra-articular anatomical landmarks, such as the medial boundary of the tibial tuberosity [16, 20], the medial third boundary of the tibial tuberosity [20, 23], the anterior cortex of the tibia [16], the anterior tibial crest [19, 21] and the tibial posterior condylar [23, 25, 26]. However, there is still a lack of consensus on tibial rotational alignment due to the low confidence and high individual variability of reference axes [13, 27].

The patellar tendon, as a continuation of the quadriceps tendon, is one of the strongest ligaments in the human body. It terminates downward at the tibial tuberosity, which can be easily identified during surgery and unaffected by knee osteoarthritis. The medial boundary of the patellar tendon at the attachment and the middle of the posterior cruciate ligament (PCL) termination constitute the Akagi line, which is considered as the most reliable and widely applied anatomical landmark [24]. However, it is often impossible to identify the posterior cruciate ligament termination after proximal tibial osteotomy. In addition, it was found that the accuracy of the Akagi line differed among the female and male [19, 23, 27].

Most of the previous studies identified the SEA on the same image on 2D-CT [11]. However, both anatomical landmarks of the SEA rarely appear on the same CT image. It is necessary to identify both anatomical landmarks on three-dimension (3D) models to decrease misevaluation. Therefore, in this study our aim was to investigate three-dimensionally: (1) whether the location of the patellar ligament attachment site can be influenced by gender difference as an anterior anatomical landmark; (2) whether the geometric center (GC) of the osteotomy plane can replace the middle of the PCL termination as the posterior anatomical landmark.

## Materials and methods

One-hundred thirty-five patients (82 women and 53 men) with varus deformities undergoing primary total knee arthroplasty at our institution from January 2021 to February 2022 were enrolled. Kellgren and Lawrence grade 3 and 4 were achieved among the participants. OA patients met the inclusion criteria only if they had taken computed tomographic (CT) scans and full-length lower extremity radiographs of the knees preoperatively. The patients with severe bone defect in tibial plateau, hemophilic arthritis, rheumatoid arthritis, or a history of knee trauma or infection were excluded. Patient demographic data including age, gender, hip-knee-ankle (HKA) angle, and body mass index (BMI, kg/m^2^) was collected and is listed in Table 1. The characteristics of female and male patients were 65.6 ± 7.9/66.2 ± 7.6 years (age), 26.1 ± 3.5/25.7 ± 3.1 kg/m^2^ (BMI) and 8.9° ± 5.6°/8.4° ± 5.0° (HKA angle), respectively. No significant differences were shown between male and female patients in terms of age, BMI and HKA angle. The research was reviewed and approved by local Medical Ethics Committee (Additional file 1).

**Table 1:** Demographic data of the female and male

Parameter	Whole patients (n = 135) Mean ± SD (range)	Female (n = 82) Mean ± SD (range)	Male (n = 53) Mean ± SD (range)	*P* value
Age (years)	65.8 ± 7.7 (43,89)	65.6 ± 7.9 (47,89)	66.2 ± 7.6 (43,86)	n. s
BMI (kg/m^2^)	26.0 ± 3.4	26.1 ± 3.5	25.7 ± 3.1	n. s
HKA angle (°)	8.7 ± 5.3 (1.3,26.0)	8.9 ± 5.6 (1.5,26.0)	8.4 ± 5.0 (1.3,23.1)	n. s

All affected lower limbs of the patients were ensured to be in neutral position and fully extended during CT scans. CT images of the knees with 1.25 mm slice thickness were imported into 3D reconstruction software (Mimics; Materialize, Leuven, Belgium) in DICOM (Digital Imaging and Communications in Medicine) format. With removing of osteophytes, 3D knee models were reconstructed in a CAD software program (Solidworks; Dassault, Massachusetts, USA). The osteotomy plane was taken 8 mm away from the center of the lateral tibial plateau containing 2 mm thickness of cartilage. This approach can guarantee 10 mm thickness of osteotomy. The osteotomy direction was perpendicular to the tibial anatomical axis with a posterior slope angle of 0°.

**Fig. 1:**
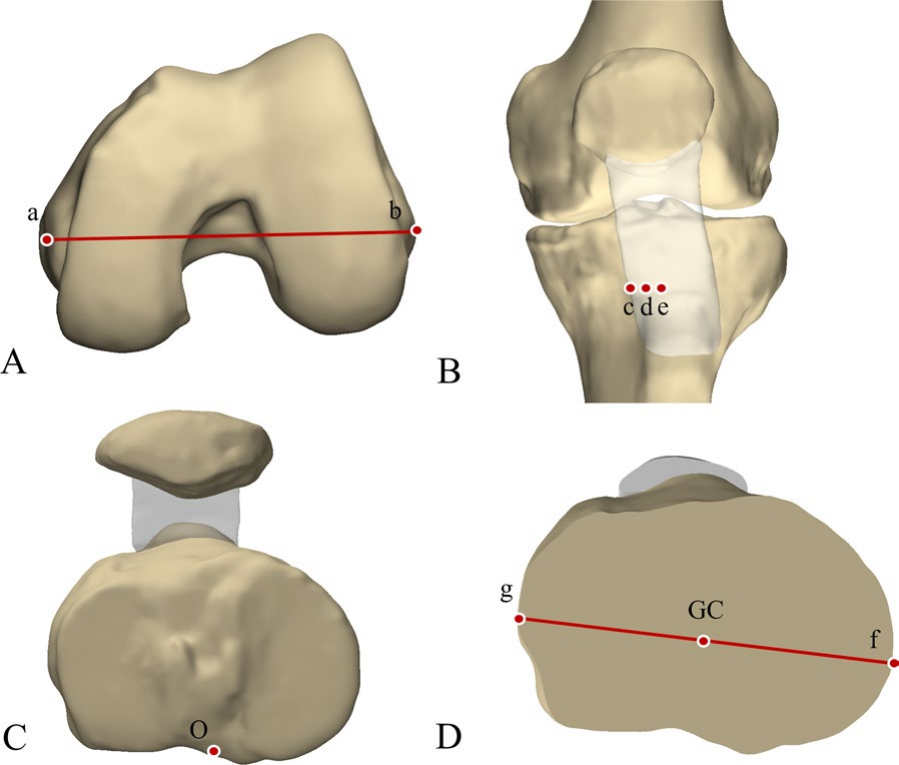
Bony landmarks for rotational alignment reference axes. **A** the surgical transepicondylar axis (a) the medial epicondylar sulcus; (b) the prominent point of lateral epicondyle. **B** (c) the medial border point of the patellar tendon at the attachment site. (d) the medial one-sixth point of the patellar tendon at the attachment site. (e) the medial third point of the patellar tendon at the attachment site. **C** (O) the midpoint of posterior cruciate ligament insertion. **D** (f) the innermost position of the tibial plateau at the resection level. (g) the outermost positions of the tibial plateau at the resection level. (GC) the geometric center of the tibial osteotomy surface

**Fig. 2:**
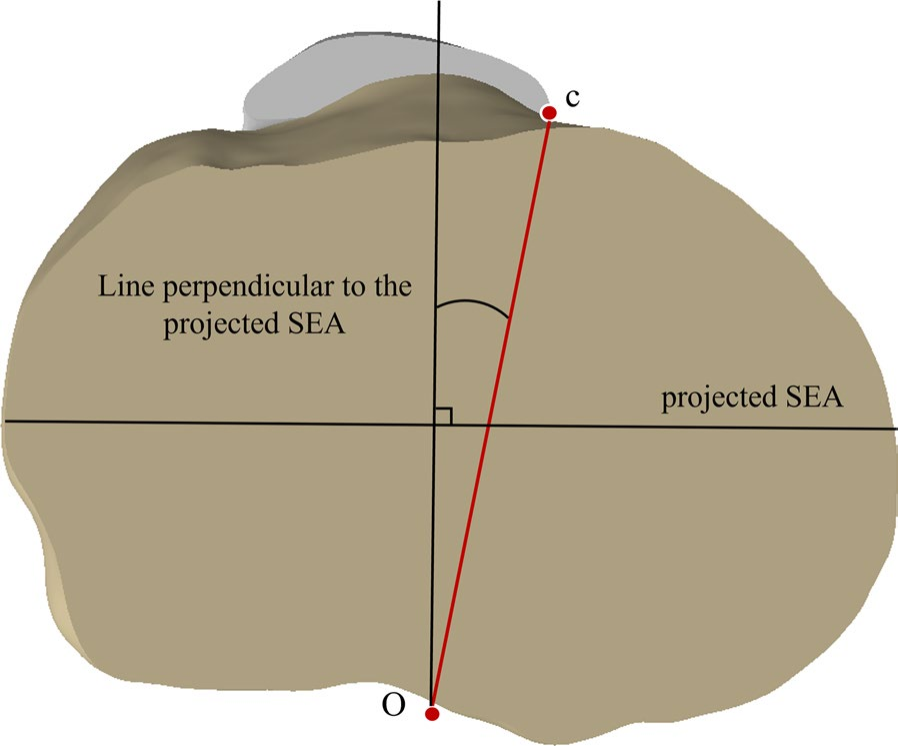
Measure rotational mismatch angles between the line perpendicular to the projected surgical transcondylar axis and tibial rotational alignment axes on the tibial osteotomy surface

**Fig. 3:**
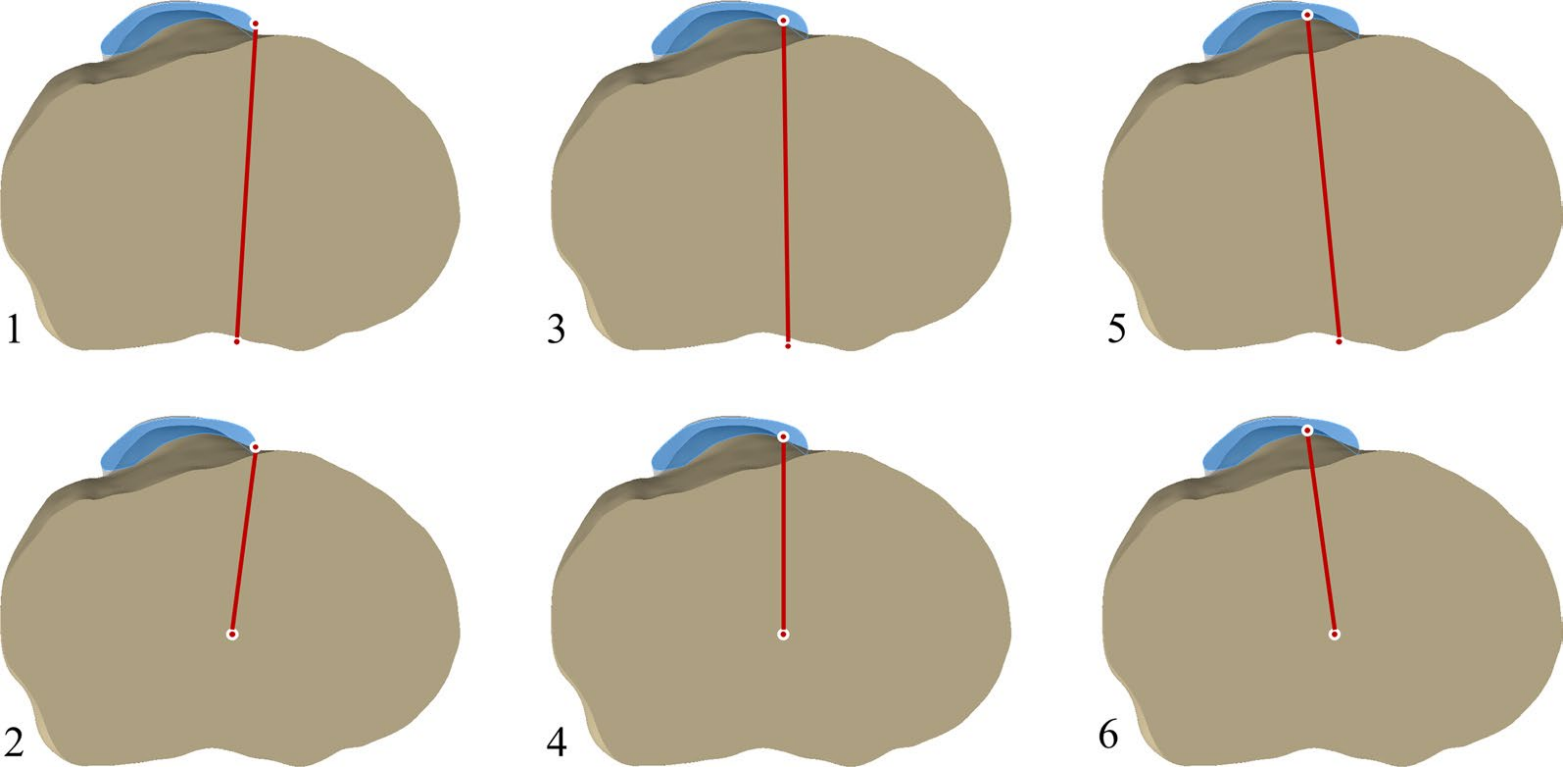
Schematic diagram of six tibial rotational alignment axes. 1: Akagi line. 2: MBPT. 3: MSPT1. 4: MSPT2. 5: MTPT1. 6: MTPT2

Each anatomical landmark was defined on the 3D models of the femur and tibia. The prominent point of lateral femoral epicondyle and the medial femoral epicondylar sulcus were marked on femoral 3D models to establish the SEA (Fig. 1A). The line perpendicular to the projection of the SEA on the tibial osteotomy surface was drawn (Fig. 2). In the tibial 3D models, five anatomical landmarks were described including three anterior anatomical landmarks (the medial boundary, the medial sixth point, and the medial third point of the patellar tendon attachment site) and two posterior anatomical landmarks (the geometric center of the tibial osteotomy plane and the middle of the PCL termination) (Fig. 1). The middle point of the line passing through the innermost and outermost positions of the resected tibial plateau was considered as the geometric center of the tibial osteotomy surface. Six tibial rotational axes were established (Fig. 3):

1. *Akagi line* the line connecting the medial boundary of the patellar tendon attachment site and the middle of the PCL termination.
2. *The medial boundary axis of the patellar tendon (MBPT)* the line connecting the medial boundary of the patellar tendon attachment site and the geometric center of the tibial osteotomy plane.
3. *The medial sixth axis of the patellar tendon 1 (MSPT1)* the line connecting the medial one-sixth of the patellar tendon attachment site and the middle of the PCL termination.
4. *The medial sixth axis of the patellar tendon 2 (MSPT2)* the line connecting the medial one-sixth of the patellar tendon attachment site and the geometric center of the tibial osteotomy plane.
5. *The medial third axis of the patellar tendon 1 (MTPT1)* the line connecting the medial one-third of the patellar tendon attachment site and the middle of the PCL termination.
6. *The medial third axis of the patellar tendon 2 (MTPT2)* the line connecting the medial one-third of the patellar tendon attachment site and the geometric center of the tibial osteotomy plane.

The internal rotational angles of tibial rotational axes relative to the vertical line of the SEA were recorded as negative values and the external rotational angles were recorded as positive values (Fig. 2). All measured angles were accurate to 0.1°. The percentage of the cases which tibial rotational axes differed by 3°, 5°, and 10° from the line perpendicular to the SEA was calculated in all models.

The mismatch angle between six tibial rotational axes and the SEA was measured by two experienced physicians who were not obtained with any patient information, respectively. The measurements were performed again 1 month after the initial measurements by one of the physicians.

## Statistical methods

Statistical analyses were performed using SPSS statistical software (version 26.0; SPSS Inc, Chicago, IL, USA). The normality assumption of the measured data was completed by the Shapiro–Wilk test. The student t test was performed to determine the significance between gender differences in 82 female and 53 male patients. The student t test was performed between the Akagi line and MBPT, between MSPT1 and MSPT2, between MTPT1 and MTPT2 which refer to the same location of the patellar tendon attachment site but different posterior anatomical landmarks. The one-way ANOVA was used to analyze the mean angular differences and variance among the six tibial rotational axes. Bonferroni multiple comparison method was performed as post hoc analysis. Probability (*P*) values < 0.05 were considered to be statistically significant.

## Results

Intra- and interobserver variability were 0.89 and 0.93, respectively, which was assessed by the intraclass correlation coefficients (ICC). The Shapiro–Wilk test showed that all measurement data were in accordance with a normal distribution.

The mean absolute deviations and standard deviations of the rotational mismatch angle of six tibial rotational axes relative to the line perpendicular to the projected SEA, are shown in Table 2. The Akagi line and the MBPT were internally rotated by 1.8° ± 5.1° and 2.5° ± 5.3° , respectively. In contrast, the MSPT1, MSPT2, MTPT1, and MTPT2 were externally rotated by 2.8° ± 5.3°, 4.5° ± 5.4°, 7.3° ± 5.4°, and 11.6° ± 5.8° , respectively (Table 2). Gender differences were found in six tibial rotational axes (*P* < 0.05). The tibial rotational axes in the female were significantly more externally rotated than that in the male (Table 2).

**Table 2:** Mismatch angles between tibial rotational alignment axes and the SEA

Parameter	Whole patients (n = 135) Mean ± SD (range)	Female (n = 82) Mean ± SD (range)	Male (n = 53) Mean ± SD (range)	*P* value
Akagi line	− 1.8 ± 5.1 (− 17.1, 10.2)	− 1.1 ± 5.1 (− 15.8, 10.2)	− 2.9 ± 5.0 (− 17.1, 6.8)	< 0.05
MBPT	− 2.5 ± 5.3 (− 19.5, 9.7)	− 1.4 ± 5.4 (− 17.4, 9.7)	− 4.1 ± 4.8 (− 19.5, 7.9)	< 0.01
MSPT1	2.8 ± 5.3 (− 12.1, 13.4)	3.5 ± 5.2 (− 12.1, 13.4)	1.6 ± 5.4 (− 12.0, 10.8)	< 0.05
MSPT2	4.5 ± 5.4 (− 14.0, 16.9)	5.6 ± 5.2 (− 11.6, 16.9)	2.9 ± 5.3 (− 14.0, 12.8)	< 0.01
MTPT1	7.3 ± 5.4 (− 8.8, 17.7)	8.1 ± 5.2 (− 8.8, 17.7)	6.2 ± 5.5 (− 7.5, 17.1)	< 0.05
MTPT2	11.6 ± 5.8 (− 6.9, 24.0)	12.8 ± 5.6 (− 4.6, 24.0)	9.7 ± 5.6 (− 6.9, 19.4)	< 0.01

The percentage of the cases which tibial rotational axes differed by 3°, 5°, and 10° from the line perpendicular to the SEA are shown in Table 3. The Akagi line showed the lowest percentage of outliers followed by MBPT, MSPT1, MSPT2, MTPT1, and MTPT2.

**Table 3:** Percentage of outliers for tibial rotational alignment axes

Parameter (angle)	3° (%)	5° (%)	10° (%)
Akagi line	60.0	36.3	5.9
MBPT	59.3	35.6	8.9
MSPT1	63.0	40.0	13.3
MSPT2	75.6	55.6	14.8
MTPT1	83.0	68.9	28.9
MTPT2	97.0	89.6	63.0

**Fig. 4:**
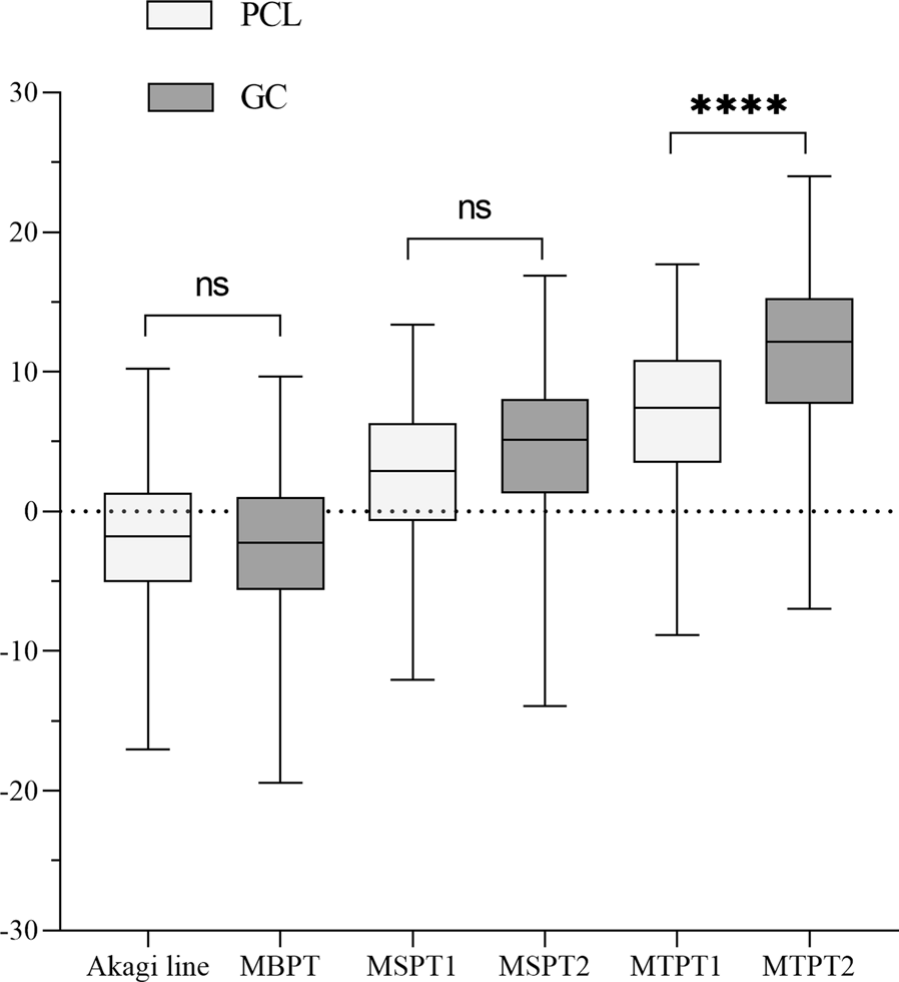
The boxplot shows rotational mismatch angles between tibial rotational alignment axes and the SEA (ns, no statistically difference; ****, *P* < 0.0001)

There was no statistical difference between the Akagi line and MBPT. The same was shown between MSPT1 and MSPT2. Statistically significant differences existed between MTPT1 and MTPT2 (Fig. 4).

## Discussion

This study found the following to be the most important results: (1) gender differences exist when choosing patellar tendon as anatomic landmarks for tibial rotational alignment in total knee arthroplasty. The female are better suited to reference the medial boundary of the patellar tendon attachment site, while the male are better suited to reference the medial one-sixth of the patellar tendon attachment site. (2) the geometric center of the tibial osteotomy plane could be used as the posterior anatomic landmark instead of the middle of the posterior cruciate ligament termination when referring to the medial boundary or medial one-sixth of the patellar tendon attachment site.

A series of anatomical reference axes have been proposed to improve the accuracy of tibial rotational alignment, in which the Akagi line has the most accurate and least variable [24]. The Akagi line refers the medial boundary of the patellar tendon attachment site as the anterior anatomic landmark and the middle of the PCL termination as the posterior anatomic landmark. The reasons why both anatomical landmarks are used as references are as follows: (1) The PCL attaches to the notch of tibial posterior condyle which is in the center of the knee and can be clearly identified as an elliptical plaque in proximal tibia on CT scans. (2) The patellar tendon can be clearly identified as an area composed of hypodense soft tissue on CT scans. The medial and lateral boundaries of its attachment site can be differentiated easily on CT scans or in TKA. (3) It was reported that the medial percentage width of the patellar tendon attachment site which was separated by the tibial anterior–posterior axis (a line passing through the middle of the PCL termination and perpendicular to the SEA in an extended knee position) at the attachment level was at an average of − 0.2% ± 10.4% in healthy people. It can be assumed that the vertical line of the SEA is basically parallel to the line connecting the middle of the PCL termination and the medial boundary of the patellar tendon attachment site.

However, the Akagi line also has its own drawback that the proximal tibial osteotomy in TKA will increases the difficulty of identification of the PCL terminations. Kawahara [15] found that the line perpendicular to the projected SEA passed through the geometrical center of the proximal tibial osteotomy surface and the medial one-sixth of the patellar tendon attachment site. It was the first time to propose to reference the medial one-sixth of the patellar tendon attachment site as an anatomical landmark for tibial rotational axis. MA [21] compared the differences between the SEA and the tibial rotational axes which were referenced to the patella tendon attachment site by reconstructing the 3D models of the knee recently. It was found that the line connecting the GC of the tibial osteotomy plane and the medial boundary or medial one-sixth of the patellar tendon attachment site could be an ideal substitute for the Akagi line for the tibial rotational alignment. The GC of the tibial osteotomy surface in that study was characterized as the center of the oval that best fits the margin on tibial resection level. In his previous study, the GC of the tibial osteotomy plane was also defined as the midpoint of the center of the circles best fitting to the edge of medial or lateral tibial plateau [20]. However, these approaches are not easily applied intraoperatively. Therefore, in this study, the GC of the tibial osteotomy surface was defined as the midpoint of the longest medial–lateral axis, which has the advantage of facilitating intraoperative definition.

The influence of gender differences on the Akagi line has been discussed in previous studies. Lu [19] found the mismatch angles between the Akagi line and the SEA were 0.8° ± 5.0° and 3.0° ± 4.5° in the non-osteoarthritis knees of the male and female, respectively. The angular differences between the male and female have also been shown to exist in the varus knees [23]. However, the influence of gender differences on the locations of the patellar tendon attachment site as the anatomical landmark has not been investigated.

As previously mentioned, the Akagi line was the most reliable rotational reference axis for tibial component in all patients. The average mismatch angle between the Akagi line and the SEA was − 1.8° ± 5.1° with the least mean absolute deviation and standard deviation. The average malrotation angle of MBPT was 2.5° ± 5.3°, which was internally rotated like the Akagi line. MSPT1, MSPT2, MTPT1, and MTPT2 were externally rotated by 2.8° ± 5.3°, 4.5° ± 5.4°, 7.3° ± 5.4°, and 11.6° ± 5.8° , respectively. However, the conclusion was not accurate when the gender difference is taken into consideration. All tibial rotation axes referring the patellar tendon site showed significant differences between the female and male. Tibial rotational axes in the female were significantly more externally rotated than those in the male. The Akagi line was still the most reliable tibial rotational axis in the female, but it was different in the male. The line with the least mean absolute deviation and variance in the male was MSPT1. It is more reliable for the male to reference the medial one-sixth of the patellar tendon attachment site than the medial boundary as the anterior anatomical landmark. It may cause internal misalignment of tibial components if the Akagi line is still seen as the standard reference axis in the male.

When comparing the tibial rotational axe that refer to the same location of the patellar tendon attachment site but different posterior anatomical landmarks, significant difference was shown between MTPT1 and MTPT2. However, there were no significant differences between the Akagi line and MBPT, and between MSPT1 and MSPT2. It is proven that the geometric center of the tibial osteotomy plane can be a substitute for the middle of the PCL termination as the posterior anatomical landmark when refer to the medial boundary or one-sixth of the patellar tendon attachment site as the posterior anatomical landmark.

This study has the following limitations: First, only the OA patients with varus knees were enrolled in the study. The influence of the varus or valgus deformity of the knees on tibial rotation alignment is not taken into consideration. Tibial osteotomy thickness is different between the varus and valgus knees. The difference can affect the shape of tibial osteotomy plane, and the accuracy of the location of the GC of the tibial osteotomy plane further. However, the cases of the male patients with valgus knees at our institution was not sufficient to meet the sample size requirement during the study period. Second, other factors except the gender differences that may have an impact on the accuracy of the anatomical landmarks for tibial rotational alignment exist. On the one hand, the position of the patellar ligament at the attachment can be influenced by the locations of the tibial tuberosity. It has been demonstrated that the TT-TG distance correlates with the accuracy of the Akagi line. On the other hand, the tibial osteotomy direction in this study only considered a posterior slope angle of 0°, but it has been demonstrated that tibial rotational axes tend to externally rotate as the posterior slope angle increases.Moreover, no consideration was given to the effect of femur or body size on tibial rotational alignment. The influences of these factors need to be further discussed. Lastly, the population of the study was limited to Chinese subjects. The anatomic differences of proximal tibia may prevent conclusions from being applied to Caucasian populations.

Nevertheless, this study provides valuable information on assessing which location of the patellar tendon at the attachment is the most suitable anatomical landmark for tibial rotational alignment in the male or female patients by means of 3D virtual surgery.

## Conclusion

When referring patellar tendon attachment site as anterior anatomical landmarks for tibial rotational alignment, the influence of gender difference on the accuracy needs to be taken into account. The geometric center of the tibial osteotomy plane can be used as a substitute for the middle of the PCL termination when reference the medial boundary or one-sixth of the patellar tendon attachment site.

## Supplementary Information

**Additional file 1:** The statement on ethics approval obtained from the Affiliated Hospital of Xuzhou Medical University (XYFY2021-KL312-01).

## Data Availability

The data will be available upon request.

## References

[1] Abdelnasser MK, Adi MM, Elnaggar AA, Tarabichi S (2020). Internal rotation of the tibial component in total knee arthroplasty can lead to extension deficit. Knee Surg Sports Traumatol Arthrosc.

[2] Asano T, Akagi M, Nakamura T (2005). The functional flexion-extension axis of the knee corresponds to the surgical epicondylar axis: in vivo analysis using a biplanar image-matching technique. J Arthroplasty.

[3] Ayers DC, Yousef M, Zheng H, Yang W, Franklin PD (2022). The prevalence and predictors of patient dissatisfaction 5-years following primary total knee arthroplasty. J Arthroplasty.

[4] Bates NA, Nesbitt RJ, Shearn JT, Myer GD, Hewett TE (2018). The influence of internal and external tibial rotation offsets on knee joint and ligament biomechanics during simulated athletic tasks. Clin Biomech (Bristol, Avon).

[5] Bedard M, Vince KG, Redfern J, Collen SR (2011). Internal rotation of the tibial component is frequent in stiff total knee arthroplasty. Clin Orthop Relat Res.

[6] Bell SW, Young P, Drury C, Smith J, Anthony I, Jones B (2014). Component rotational alignment in unexplained painful primary total knee arthroplasty. Knee.

[7] Berger RA, Crossett LS, Jacobs JJ, Rubash HE (1998). Malrotation causing patellofemoral complications after total knee arthroplasty. Clin Orthop Relat Res.

[8] Bourne RB, Chesworth BM, Davis AM, Mahomed NN, Charron KD (2010). Patient satisfaction after total knee arthroplasty: who is satisfied and who is not?. Clin Orthop Relat Res.

[9] Cerquiglini A, Henckel J, Hothi H, Rotigliano N, Hirschmann MT, Hart AJ (2018). 3D patient imaging and retrieval analysis help understand the clinical importance of rotation in knee replacements. Knee Surg Sports Traumatol Arthrosc.

[10] Cho BW, Hong HT, Koh YG, Choi J, Park KK, Kang KT (2021). Analysis of gender differences in the rotational alignment of the distal femur in kinematically aligned and mechanically aligned total knee arthroplasty. J Clin Med.

[11] De Valk EJ, Noorduyn JC, Mutsaerts EL (2016). How to assess femoral and tibial component rotation after total knee arthroplasty with computed tomography: a systematic review. Knee Surg Sports Traumatol Arthrosc.

[12] Fottner A, Woiczinski M, Schroder C, Schmidutz F, Weber P, Muller PE (2020). Impact of tibial baseplate malposition on kinematics, contact forces and ligament tensions in TKA: a numerical analysis. J Mech Behav Biomed Mater.

[13] Howell SM, Chen J, Hull ML (2013). Variability of the location of the tibial tubercle affects the rotational alignment of the tibial component in kinematically aligned total knee arthroplasty. Knee Surg Sports Traumatol Arthrosc.

[14] Huang CH, Lu YC, Hsu LI, Liau JJ, Chang TK, Huang CH (2020). Effect of material selection on tibial post stresses in posterior-stabilized knee prosthesis. Bone Jt Res.

[15] Kawahara S, Okazaki K, Matsuda S, Mitsuyasu H, Nakahara H, Okamoto S, Iwamoto Y (2014). Medial sixth of the patellar tendon at the tibial attachment is useful for the anterior reference in rotational alignment of the tibial component. Knee Surg Sports Traumatol Arthrosc.

[16] Kim JI, Jang J, Lee KW, Han HS, Lee S, Lee MC (2017). Anterior tibial curved cortex is a reliable landmark for tibial rotational alignment in total knee arthroplasty. BMC Musculoskelet Disord.

[17] Klasan A, Twiggs JG, Fritsch BA, Miles BP, Heyse TJ, Solomon M (2020). Correlation of tibial component size and rotation with outcomes after total knee arthroplasty. Arch Orthop Trauma Surg.

[18] Kobayashi H, Akamatsu Y, Kumagai K, Kusayama Y, Aratake M, Saito T (2015). Is the surgical epicondylar axis the center of rotation in the osteoarthritic knee?. J Arthroplasty.

[19] Lu Y, Ren X, Liu B, Xu P, Hao Y (2020). Tibiofemoral rotation alignment in the normal knee joints among Chinese adults: a CT analysis. BMC Musculoskelet Disord.

[20] Ma Y, Mizu-Uchi H, Okazaki K, Ushio T, Murakami K, Hamai S (2018). Effects of tibial baseplate shape on rotational alignment in total knee arthroplasty: three-dimensional surgical simulation using osteoarthritis knees. Arch Orthop Trauma Surg.

[21] Ma Y, Mizu-Uchi H, Ushio T, Hamai S, Akasaki Y, Murakami K (2019). Bony landmarks with tibial cutting surface are useful to avoid rotational mismatch in total knee arthroplasty. Knee Surg Sports Traumatol Arthrosc.

[22] Merican AM, Ghosh KM, Iranpour F, Deehan DJ, Amis AA (2011). The effect of femoral component rotation on the kinematics of the tibiofemoral and patellofemoral joints after total knee arthroplasty. Knee Surg Sports Traumatol Arthrosc.

[23] Nam JH, Koh YG, Kim PS, Kim G, Kwak YH, Kang KT (2020). Evaluation of tibial rotational axis in total knee arthroplasty using magnetic resonance imaging. Sci Rep.

[24] Saffarini M, Nover L, Tandogan R, Becker R, Moser LB, Hirschmann MT (2019). The original Akagi line is the most reliable: a systematic review of landmarks for rotational alignment of the tibial component in TKA. Knee Surg Sports Traumatol Arthrosc.

[25] Sahin N, Atici T, Ozturk A, Ozkaya G, Ozkan Y, Avcu B (2012). Accuracy of anatomical references used for rotational alignment of tibial component in total knee arthroplasty. Knee Surg Sports Traumatol Arthrosc.

[26] Sunnassee Y, Zhang H, Southern EP, Wang Y, Shen Y (2015). Reliability of intra-articular rotational axes at standard tibial resection level and effect of resecting distally. J Knee Surg.

[27] Yike D, Tianjun M, Heyong Y, Chongyang X, Hongrui Z, Ai G (2021). Different rotational alignment of tibial component should be selected for varied tibial tubercle locations in total knee arthroplasty. Knee Surg Sports Traumatol Arthrosc.

