# Supplementary material for: Gender differences affect the location of the patellar tendon attachment site for tibial rotational alignment in total knee arthroplasty
Source: J Orthop Surg Res. 2022 Jul 26;17:362. doi: 10.1186/s13018-022-03248-5 (PMC9327362; doi:10.1186/s13018-022-03248-5)
Supplement: Supplementary file 1 — Additional file 1: The statement on ethics approval obtained from the Affiliated Hospital of Xuzhou Medical University (XYFY2021-KL312-01). [file 13018_2022_3248_MOESM1_ESM.pdf]

徐州医科大学附属医院医学伦理委员会

伦理审查意见

意见号	XYFY2021-KL312-01				
项目名称	关节置换三维术前规划与临床应用				
研究机构	徐州医科大学附属医院				
研究科室	骨科	主要研究者	陈向阳	职称	主任医师
审查类别	科研立项		审查方式	快审	
审查日期	2021-12-20、2021-12-23		审查地点	委员办公室	
申办者名称	徐州医科大学附属医院				
审查文件	1 伦理审查申请表<br>2 研究方案(V1.0/2021.03.08)<br>3 知情同意书 (V1.0/2021.03.08)<br>4 研究经济利益声明<br>5 主要研究者履历、研究人员名单及研究职责				

审查意见

遵循卫生部《涉及人的生物医学研究伦理审查办法)》(2016)、SFDA《药物临床试验质量管理规范》(2020)、《医疗器械临床试验质量管理规范》(2016)、《医疗技术临床应用管理办法》(2018)、WMA《赫尔辛基宣言》和 CIOMS《人体生物医学研究国际道德指南》及临床试验管理规范指导原则（ICH-GCP）的伦理原则，经本伦理委员会审查，**同意按所审查的临床研究方案、知情同意书开展本项研究。**

请遵循 GCP 原则、遵循 ICH-GCP 指导原则、遵循伦理委员会批准的方案开展临床研究，保护受试者的健康与权利。

伦理委员会对该批准项目进行跟踪审查直至研究结束，具体要求为：

①研究过程中若变更主要研究者，对临床研究方案、知情同意书、招募材料等的任何修改，请申请人提交修正案审查申请。

②请按照伦理委员会规定的年度/定期跟踪审查频率，申请人在截止日期前1个月提交研究进展报告；申办者应当向组长单位伦理委员会提交各中心研究进展的汇总报告；当出现任何可能显著影响试验进行、或增加受试者危险的情况时，请申请人及时向伦理委员会提交书面报告。

③发生严重不良事件及非预期药物不良反应，请申请人及时向伦理委员会提交书面报

告。<br>④研究纳入了不符合纳入标准或符合排除标准的受试者，符合中止试验规定而未让受试者退出研究，给予错误治疗或剂量，给予方案禁止的合并用药等没有遵从方案开展研究的情况或可能对受试者的权益/健康以及研究的科学性造成不良影响等违背 GCP 原则的情况，请申办者/监查员/研究者提交违背方案报告。<br>⑤申请人暂停或提前终止临床研究，请及时提交暂停/终止研究报告。<br>⑥完成临床研究，请申请人提交研究完成报告。	
年度/定期跟踪审查频率	1年
有效期	2年
主任委员签名	许铁
签发日期	2021.12.27
伦理委员会	徐州医科大学附属医院医学伦理委员会（盖章）
地址：徐州市淮海西路99号，邮编221000，联系人：翟凤平 ，联系电话：0516-85802291	
